# Regression-Based Docking System for Autonomous Mobile Robots Using a Monocular Camera and ArUco Markers

**DOI:** 10.3390/s25123742

**Published:** 2025-06-15

**Authors:** Jun Seok Oh, Min Young Kim

**Affiliations:** 1 School of Electronic and Electrical Engineering, Kyungpook National University, Daegu 41566, Republic of Korea; 2025006910@knu.ac.kr; 2 School of Electronics Engineering, Kyungpook National University, Daegu 41566, Republic of Korea; 3 Research Center for Neurosurgical Robotic Systems, Kyungpook National University, Daegu 41566, Republic of Korea; 4 KNU–LG Electronics Convergence Research Center, Kyungpook National University, Daegu 41566, Republic of Korea

**Keywords:** monocular camera, ArUco markers, regression model, autonomous docking

## Abstract

This paper introduces a cost-effective autonomous charging docking system that utilizes a monocular camera and ArUco markers. Traditional monocular vision-based approaches, such as SolvePnP, are sensitive to viewing angles, lighting conditions, and camera calibration errors, limiting the accuracy of spatial estimation. To address these challenges, we propose a regression-based method that learns geometric features from variations in marker size and shape to estimate distance and orientation accurately. The proposed model is trained using ground-truth data collected from a LiDAR sensor, while real-time operation is performed using only monocular input. Experimental results show that the proposed system achieves a mean distance error of 1.18 cm and a mean orientation error of 3.11°, significantly outperforming SolvePnP, which exhibits errors of 58.54 cm and 6.64°, respectively. In real-world docking tests, the system achieves a final average docking position error of 2 cm and an orientation error of 3.07°, demonstrating that reliable and accurate performance can be attained using low-cost, vision-only hardware. This system offers a practical and scalable solution for industrial applications.

## 1. Introduction

Logistics automation has emerged as a key factor in enhancing operational efficiency and reducing costs in modern manufacturing. Autonomous mobile robots (AMRs) play a central role in enabling such automation by autonomously transporting goods within warehouses and production facilities, thereby increasing productivity and flexibility [1]. In these systems, environmental perception is typically achieved using high-precision sensors such as LiDAR or depth cameras, which support not only autonomous navigation but also automatic docking (charging connection).

However, although such sensors offer high spatial accuracy, their cost presents a significant economic burden when deploying multiple robots in large-scale industrial environments [2]. To address this issue, low-cost transport robots equipped solely with monocular cameras have recently gained attention. These systems can perform autonomous navigation along predefined AMR routes without relying on high-precision sensors, thereby improving the scalability and applicability of logistics automation systems [3].

Recent studies have also investigated advanced path planning algorithms to enhance AMR efficiency in dynamic environments. For example, multi-robot cooperative optimization approaches have been proposed for congestion-aware coverage [4], and lightweight planning strategies for indoor navigation with low-cost sensors have gained attention [5,6]. These studies highlight the need for efficient, scalable solutions in real-world logistics environments.

**Figure 1:**
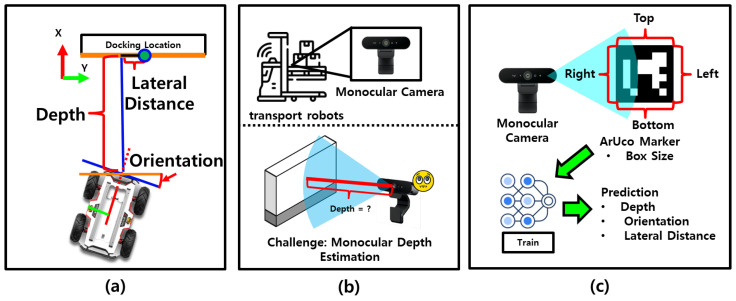
Overview of vision-based perception for docking. (**a**) Required information for docking, including depth, lateral offset, and orientation. (**b**) Robots equipped with monocular cameras lack direct access to this information. (**c**) The proposed system estimates relative pose—depth, orientation, and lateral offset—using visual features and regression models.

Nevertheless, monocular cameras have a fundamental limitation: they cannot directly measure spatial relationships such as distance, orientation, or the relative position between the robot and the charger. As shown in Figure 1a, successful docking requires accurate information on depth, lateral offset, and relative orientation between the robot and the docking station. However, in cost-sensitive industrial settings where transport robots are equipped only with monocular cameras (Figure 1b), acquiring this information directly is difficult. This limitation hinders precise autonomous docking. One potential solution is to introduce visual cues such as ArUco markers. Figure 1c illustrates the proposed system, which estimates the robot’s relative pose—depth, orientation, and lateral offset—by learning the visual changes between the robot and the docking station using a regression model trained exclusively on monocular image inputs.

A widely used method for pose estimation using ArUco markers is the SolvePnP algorithm. This algorithm estimates the camera’s position and orientation based on the observed size of the marker—which varies with the viewing angle—under the assumption that the actual size of the marker is known [7]. However, this method presents several limitations.

First, due to the inherent limitations of monocular cameras, the accuracy of depth estimation decreases significantly as the distance between the camera and the marker increases [8]. Second, when the marker is viewed from an oblique orientation, perspective distortion reduces the accuracy of pose estimation [9]. Third, SolvePnP is sensitive to environmental factors such as lighting variations and image noise, which may result in unstable marker detection. Most critically, because SolvePnP does not directly measure depth, its distance estimation is generally less reliable, and even small calibration errors can significantly affect the final outcome.

To address these limitations, this paper proposes a regression-based docking system that enables precise docking using only a monocular camera. The proposed system learns the relationship between the apparent size of the ArUco marker and the robot’s relative position and orientation by training on ground-truth distance and orientation data collected using a LiDAR sensor.

The LiDAR is used exclusively during the training phase, and the trained model operates solely with monocular input, ensuring both cost-efficiency and practical deployability. Furthermore, by leveraging a regression model based on geometric transformations of the ArUco marker, the system can simultaneously estimate both depth and orientation. The performance of the proposed system is evaluated through quantitative comparisons with the SolvePnP algorithm, demonstrating superior accuracy and robustness compared to traditional monocular vision-based pose estimation methods.

## 2. Overview of the Proposed Method

**Figure 2:**
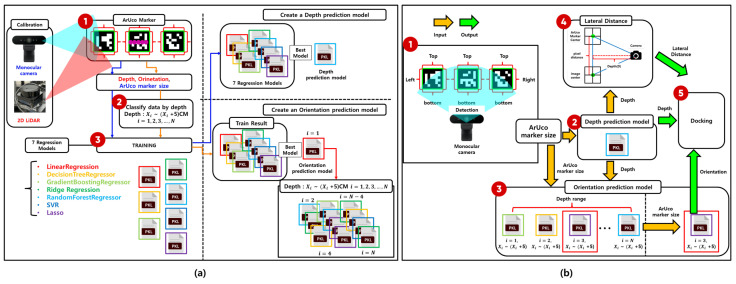
Process flow of depth and orientation prediction and docking using the trained model. (**a**) Training stage: Synchronized data from a monocular camera and 2D LiDAR are used to train the regression models. (**b**) Inference stage: The trained models predict depth, orientation, and lateral offset using only the monocular camera for docking.

Figure 2 illustrates the overall workflow of the proposed system, where (a) depicts the process of training the regression models, and (b) represents the docking procedure using the trained models.

In Figure 2a, synchronized data are collected using both a monocular camera and a 2D LiDAR sensor. The ArUco marker is detected in images captured by the monocular camera, and the 2D LiDAR scan data are projected onto the corresponding marker location to obtain accurate ground-truth values for depth and orientation. These values serve as training labels for the regression models. The monocular camera captures the marker from various distances and orientations, collecting data that reflect changes in the apparent size and shape of the marker. By combining visual features with spatial measurements, the system learns the precise relationship between the marker’s appearance and its spatial attributes—depth and orientation.

Seven regression algorithms are employed for model training: linear regression, decision tree regressor, gradient boosting regressor, ridge regression, random forest regressor, support vector regressor (SVR), and lasso regression [10,11,12,13,14,15,16]. The model demonstrating the best performance is selected based on the mean squared error (MSE) and prediction stability on the validation dataset. A single regressor is used for depth prediction, while a multi-regressor structure is adopted for orientation prediction, with specific regressors selected according to distance ranges to address geometric distortion caused by varying viewpoints.

Figure 2b illustrates the real-time inference stage. The ArUco marker is detected using the monocular camera, and its size and shape features are extracted. These features are input into the depth prediction model to estimate the distance between the robot and the marker. Based on the estimated depth, the appropriate orientation prediction model is selected and used to calculate the orientation. Additionally, the estimated depth is used to compute the lateral offset via triangulation. Ultimately, the system obtains three key parameters—depth, orientation, and lateral offset—required for precise docking and proceeds with the docking maneuver accordingly.

## 3. Proposed Depth and Orientation Estimation Concept

### 3.1. Depth Estimation from a Monocular Camera Using Marker Size and Regression

**Figure 3:**
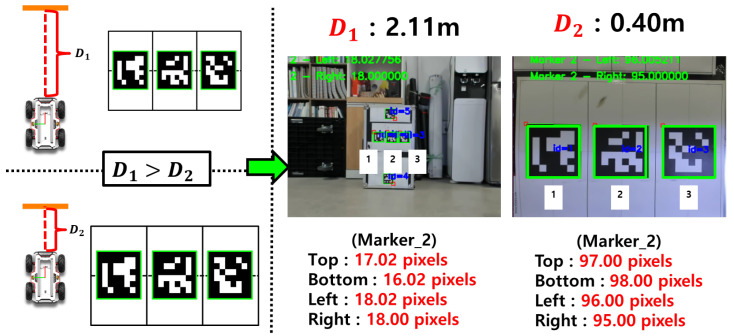
Change in the observed size of the ArUco marker in the image with respect to variations in the camera-to-marker distance.

We propose a method for indirectly estimating depth by leveraging the projection distortion characteristics of a monocular camera. As shown in Figure 3, the observed size of the ArUco marker in the image changes nonlinearly with the depth between the camera and the marker. By learning this relationship through regression models, the system can effectively predict depth using only the marker size as input.

### 3.2. Orientation Estimation from a Monocular Camera Using Marker Shape and Regression

**Figure 4:**
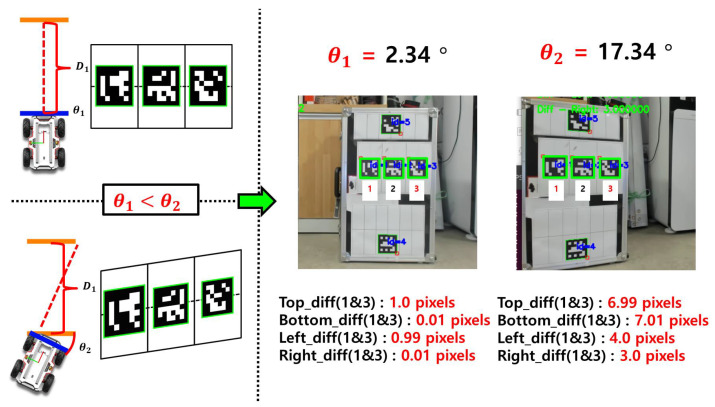
Distortion of the ArUco marker appearance due to changes in viewing orientation.

This study proposes a method for indirectly estimating orientation by leveraging the projection distortion characteristics of monocular vision. As shown in Figure 4, the appearance of the ArUco marker in the image is distorted depending on the viewing orientation. By training a regression model on this nonlinear relationship, the system can predict orientation using only the shape information of the marker.

### 3.3. Segmented Regression Approach to Address Depth–Orientation Interference

**Figure 5:**
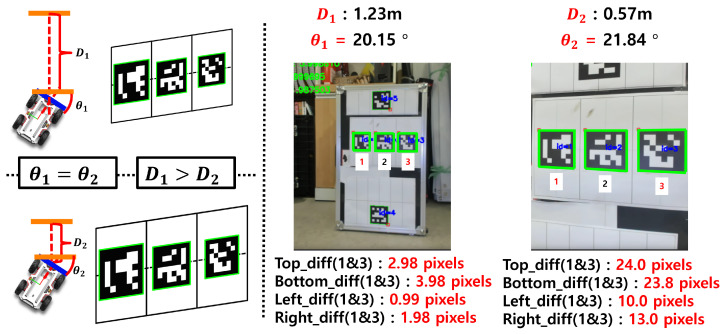
Difficulty in orientation estimation due to variations in marker distortion at different depths under the same pose.

A key challenge in orientation estimation is that, although changes in the marker shape caused by angular variations can be detected at a fixed depth, the same angular variation produces different distortions at varying depths. As shown in Figure 5, this phenomenon complicates accurate orientation estimation when depth information is not taken into account.

**Figure 6:**
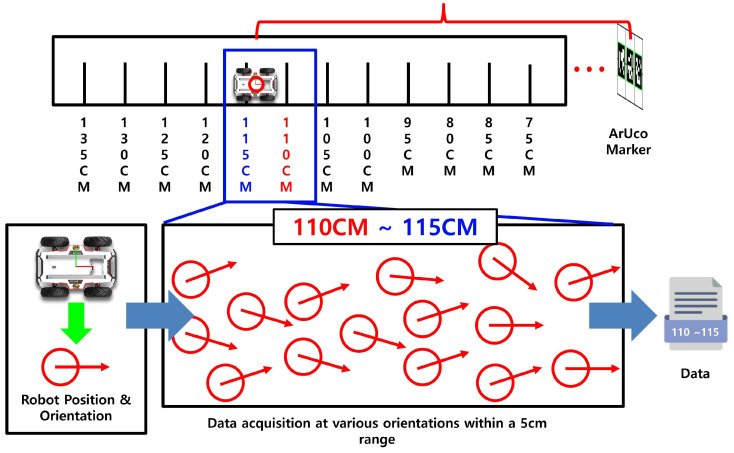
Depth segmentation at 5 cm intervals and training of individual regression models for orientation estimation within each segment.

To resolve this issue, the system decouples the effects of depth and orientation variations, as shown in Figure 6. Specifically, the system segments the depth information into fixed intervals of 5 cm. Within each depth range, pose data are collected, and individual regression models are trained for each segment. This approach minimizes the influence of distance variations and improves the precision of orientation estimation.

## 4. Model Training for Depth and Orientation Prediction

### 4.1. Sensor Calibration and Ground-Truth Acquisition for Regression Model Training

The proposed system predicts the distance and orientation between the robot and the charging station using a pre-trained regression model. To train a highly accurate model, it is essential to collect a sufficient amount of training data along with reliable ground-truth values. However, because a monocular camera alone cannot precisely measure the distance and orientation to the docking target, the system incorporates a 2D LiDAR sensor capable of high-precision distance measurement.

To effectively fuse monocular camera images with LiDAR-based depth data, a calibration process is required to align the coordinate systems of the two sensors. This involves computing both intrinsic and extrinsic parameters. The 3D coordinates (X,Y,Z) obtained from the LiDAR are projected onto the 2D image coordinates (u,v) of the monocular camera, as described in Equation (1).

**Figure 7:**
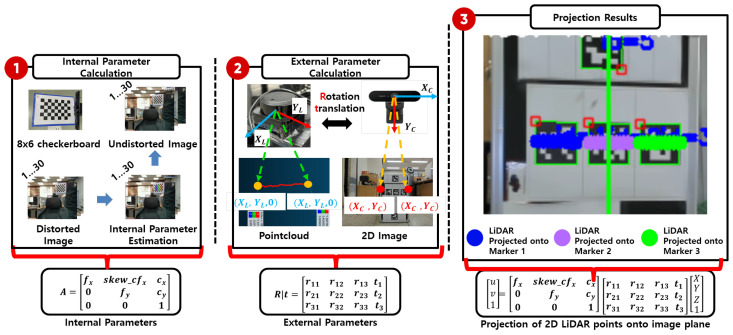
Computation of intrinsic and extrinsic calibration parameters between the 2D LiDAR and monocular camera, and projection of LiDAR data onto the image plane.

As shown in Figure 7, the intrinsic parameters are computed using images of an 8×6 checkerboard pattern captured from various positions. The extrinsic parameters (rotation and translation) are estimated using OpenCV’s SolvePnP algorithm when both the monocular camera and LiDAR observe the same object. Using the computed intrinsic matrix *K* and extrinsic parameters [R|t], the 3D coordinates (X,Y,Z) from the LiDAR are projected onto the 2D image coordinates (u,v) of the monocular camera as follows:

(1) $$\left[\begin{array}{c} u\\ v\\ 1 \end{array}\right]∼\left[\begin{array}{ccc} f_{x} & 0 & c_{x}\\ 0 & f_{y} & c_{y}\\ 0 & 0 & 1 \end{array}\right]\cdot \left[\begin{array}{cccc} r_{11} & r_{12} & r_{13} & t_{x}\\ r_{21} & r_{22} & r_{23} & t_{y}\\ r_{31} & r_{32} & r_{33} & t_{z} \end{array}\right]\cdot \left[\begin{array}{c} X\\ Y\\ Z\\ 1 \end{array}\right]$$

### 4.2. Development of Distance and Orientation Prediction Models Using Regression

**Figure 8:**
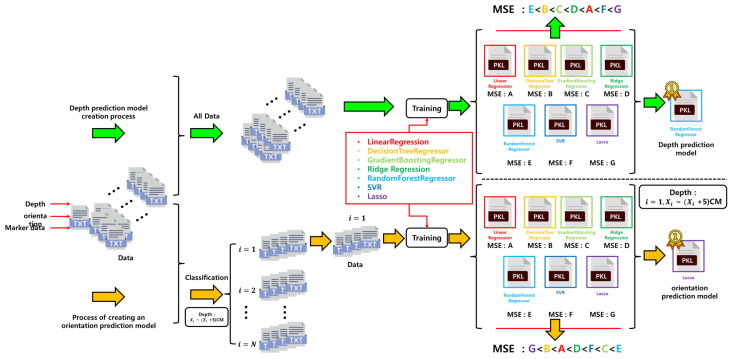
Flowchart of depth and orientation prediction model generation.

Figure 8 illustrates the overall training process for the depth and orientation prediction models. These models are trained using the geometric features of ArUco markers obtained from a monocular camera, along with the corresponding ground-truth depth and orientation values measured simultaneously by a 2D LiDAR sensor. A total of seven regression models are evaluated in this process.

For depth prediction, a single global regression model is trained, as the relationship between marker size and depth is relatively simple and consistent. In contrast, orientation prediction is more complex due to the nonlinear distortion of marker shapes at varying distances. To address this, the entire depth range (25–250 cm) is divided into 5 cm intervals, and a separate model is trained for each segment.

Because the choice of regression architecture greatly influences prediction performance, this study evaluates seven widely used models: linear regression, ridge regression, lasso regression, decision tree regressor, random forest regressor, support vector regression (SVR), and gradient boosting regressor [10,11,12,13,14,15,16]. All models are trained under the same conditions, with a maximum of 10 training iterations per model, and the model with the lowest mean squared error (MSE) is selected as the final predictor for each segment.

As a result, a total of 315 models are trained—one for depth prediction and 314 for orientation prediction (45 segments × 7 models)—and the best-performing model is selected for each segment. This approach enables the system to maintain high generalization performance and prediction accuracy across a wide range of distances.

This segmented modeling strategy is particularly effective when the number of samples and their distribution vary across depth ranges, and when the relationship between input features and targets changes due to marker distortion. In cases where some depth intervals lack sufficient data due to occlusions or limited observation conditions, linear interpolation and the use of data from adjacent intervals are applied to ensure dataset continuity and adequate training samples.

### 4.3. Performance Analysis of the Depth Prediction Model

**Figure 9:**
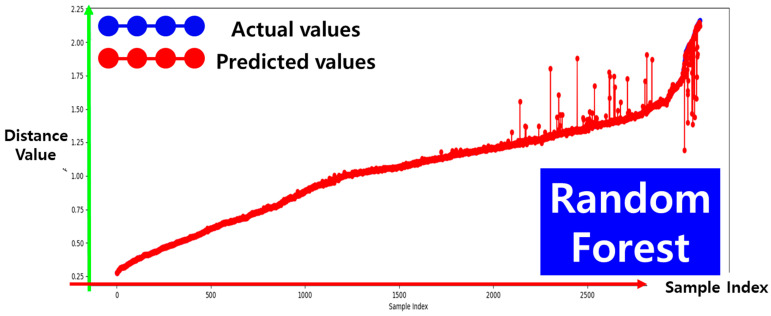
Performance evaluation results of the depth prediction model.

As shown in Figure 9, the performance evaluation of the depth prediction models indicates that the random forest regressor yields the best results. Specifically, this model achieves a mean squared error (MSE) of 0.0009 and a coefficient of determination ($R^{2}$) of 0.9937. These values indicate that the prediction error is extremely low, demonstrating a high level of accuracy in depth estimation.

To support this observation quantitatively, Table 1 presents the detailed performance metrics (MSE and $R^{2}$) of all seven regression models evaluated in this study. Decision tree and gradient boosting regressors also demonstrated strong performance, whereas linear, ridge, and lasso regressors showed comparatively lower accuracy. Among them, the random forest model, highlighted in yellow in the table, achieved the best performance in depth prediction.

### 4.4. Performance Analysis of the Orientation Prediction Model

**Figure 10:**
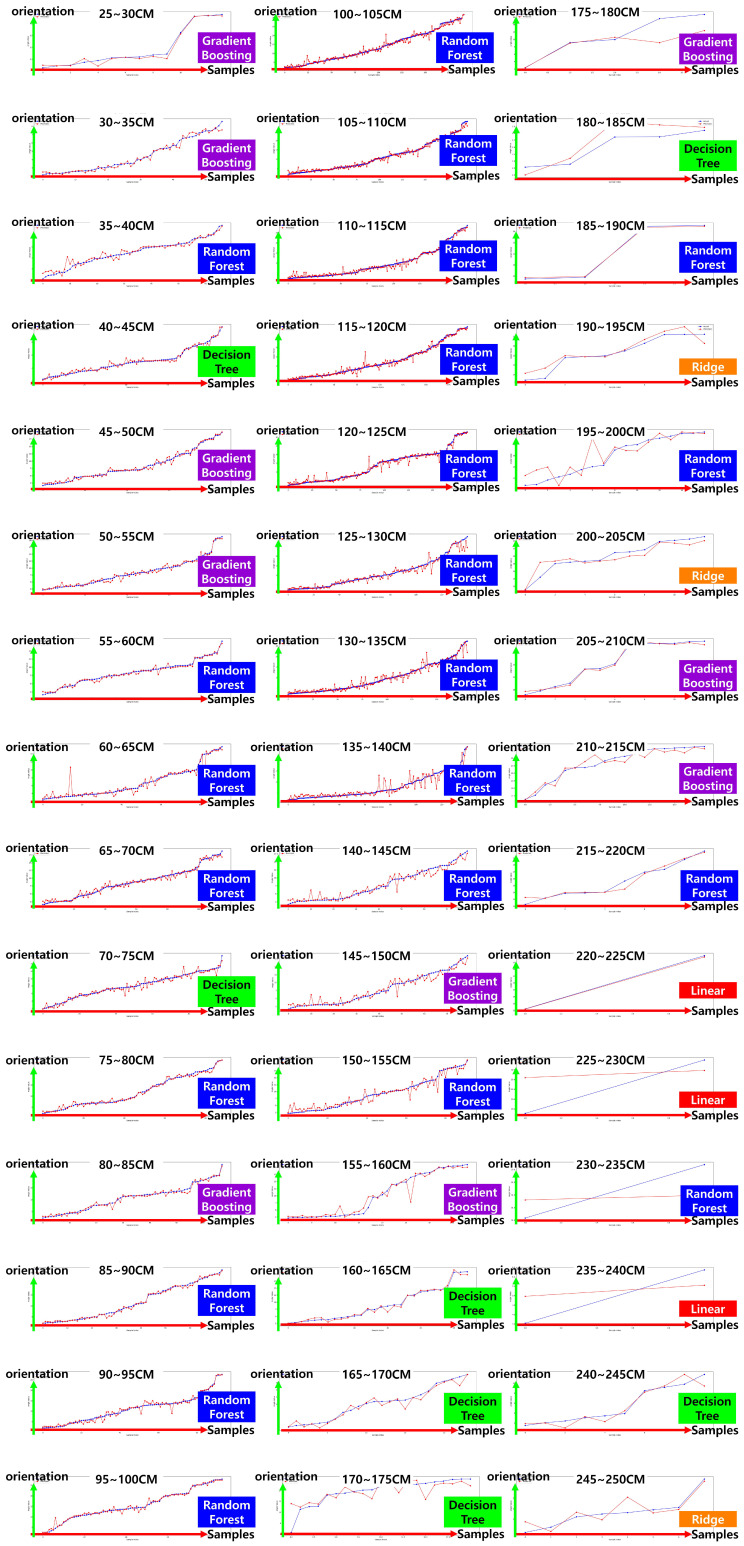
Test results of orientation prediction models. The x-axis represents the sample number (*N*), and the y-axis represents the orientation (°). The blue line indicates the ground-truth orientation, while the red line shows the orientation estimated by the model.

Figure 10 visually illustrates the performance distribution of various regression models across different distance intervals, clearly indicating that the optimal model changes depending on the distance. Notably, it is repeatedly observed that even adjacent intervals yield different best-performing models, implying that a single regression model is insufficient to effectively generalize across the entire distance range.

Table 2 summarizes the regression model with the lowest mean squared error (MSE) in each distance interval, along with the corresponding performance metrics, thereby providing empirical support for the proposed segmented model training strategy. For instance, in the 25–30 cm interval, the gradient boosting regressor achieved the best performance with an MSE of 0.0099 and a coefficient of determination ($R^{2}$) of 0.9847. In contrast, the random forest regressor exhibited consistent and robust accuracy across mid- to long-range intervals. Remarkably, even in closely spaced intervals such as 140–145 cm, 145–150 cm, and 155–160 cm, the optimal model varied between decision tree, random forest, and gradient boosting regressors, respectively.

These variations in optimal model selection across narrow distance ranges are not merely due to differences in model complexity, but rather stem from the sensitivity of ArUco marker appearance to distance-dependent visual changes. Features such as marker size, edge clarity, and position in the image can vary significantly with just a few centimeters of distance, due to factors like resolution loss, distortion, or changes in the viewing angle. Consequently, the statistical distribution of input features differs by segment, and each model’s structural characteristics (e.g., linearity, splitting criteria, or boosting mechanics) affect its ability to adapt to these variations.

Based on these observations, this study implements a segmented regression approach in which a distinct model is trained for each distance interval. This strategy significantly improves prediction accuracy, achieving an average MSE of 1.5185 and a coefficient of determination $R^{2}=0.915$ across all distances, thereby demonstrating strong generalization performance.

Additionally, Table 3 presents a comparison of MSE values for seven regression models in the 140–165 cm range, quantitatively showing that noticeable performance differences persist even between neighboring intervals. These findings reinforce that variations in marker distortion and feature quality across distances have a substantial impact on prediction accuracy, and that a single model cannot sufficiently account for such complexities.

### 4.5. Existing Monocular Depth and Orientation Estimation Method: SolvePnP

To ensure a fair performance comparison under identical sensor conditions, SolvePnP was selected as the baseline method in this study. As a widely used pose estimation algorithm that operates solely on monocular camera input, it shares the same input constraints as the proposed system, making it an appropriate benchmark. SolvePnP is commonly used in applications such as robotic vision, augmented reality (AR), and industrial automation.

SolvePnP solves the perspective-n-point (PnP) problem by using the 2D coordinates of the marker’s corners in the image and their corresponding 3D coordinates in the real world to estimate the camera’s position and orientation. This enables six-degrees-of-freedom (6-DoF) pose estimation from a single image. In this study, ArUco markers with predefined size and 3D geometry were used as visual references for implementing the SolvePnP process.

**Figure 11:**
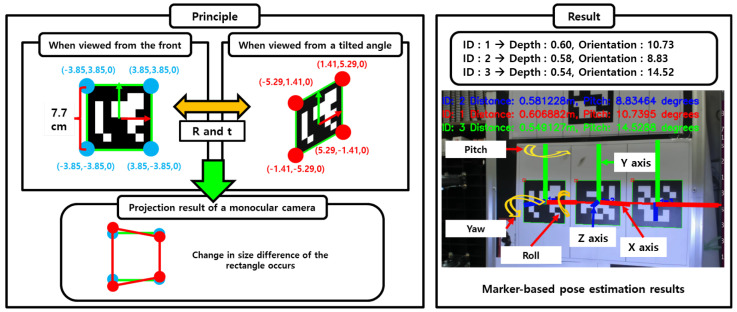
Pose estimation using SolvePnP. The algorithm estimates the six-degrees-of-freedom (6-DoF) pose of the camera relative to the marker based on the known size and 3D coordinates of the marker and the corresponding 2D image coordinates.

Figure 11 illustrates the operating principle and output of the SolvePnP algorithm. The left side shows how the projection of the marker changes under frontal and oblique views, and how pose estimation is performed based on the resulting distortion of corner positions. The right side presents the actual estimation results, including predicted distance and pitch orientation, with the projected 3D coordinate axes (roll, pitch, yaw) overlaid on the input image. This demonstrates the algorithm’s ability to estimate both position and orientation using only a monocular camera.

Despite its advantages, SolvePnP exhibits several limitations in real-world applications. Its depth estimation accuracy degrades significantly as the distance between the camera and the marker increases. Furthermore, perspective distortion caused by oblique viewing orientations reduces the accuracy of orientation estimation. The algorithm is also sensitive to environmental factors such as lighting variations and image noise, which can lead to unstable marker detection. Most importantly, because SolvePnP estimates depth geometrically rather than through direct measurement, even small errors in camera calibration or corner detection can significantly affect the final pose estimation result.

### 4.6. Comparison of Depth Estimation Performance Between the Proposed System and Existing Methods

To evaluate the effectiveness of the proposed system, a depth estimation performance comparison was conducted against the baseline method, SolvePnP. As both approaches utilize only a monocular camera, this comparison ensures a fair evaluation under identical sensor conditions. LiDAR-based measurements were used as the ground-truth reference values.

**Figure 12:**
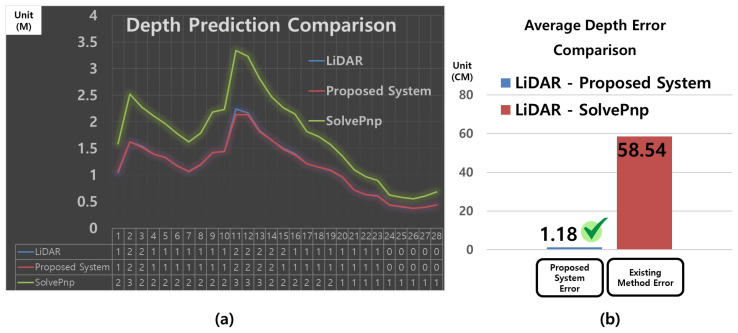
Comparison of depth prediction performance between the proposed system and SolvePnP. (**a**) Time-series depth prediction results (line graph): Depth values (*y*-axis, in meters) over sample indices (*x*-axis) for LiDAR ground truth (blue), proposed system (red), and SolvePnP (green). (**b**) Mean error (ME) comparison (bar graph): Comparison of depth prediction errors relative to LiDAR measurements, with the proposed system (blue bar) and SolvePnP (red bar).

Figure 12a visualizes the sample-wise depth prediction results presented in Table 4, showing the ground-truth values (A), the predictions from the proposed system (B), and the results from the conventional SolvePnP method (C) in a line graph. The x-axis represents the sample index, and the y-axis indicates the depth value in meters (m). The blue line corresponds to the actual depth measured by LiDAR (ground truth, A), the red line represents the predictions from the proposed system (B), and the green line indicates the SolvePnP predictions (C). As observed in the graph, the proposed system closely follows the ground-truth depth values with stable accuracy, while SolvePnP exhibits large deviations from the actual values and shows inconsistent performance.

Figure 12b presents a bar chart of the average errors computed from the same table (|A–B| and |A–C|). The blue bar (“Proposed System Error”) shows an average error of 1.18 m between the proposed system and the LiDAR ground truth, whereas the red bar (“Existing Method Error”) indicates a much larger mean error of 58.54 m for the SolvePnP predictions.

The raw data used to generate Figure 12 are summarized in Table 4. The table shows that SolvePnP performs relatively well at short distances below approximately 1.0 m (e.g., indices 20–28, with errors ranging from 0.18 to 0.40 m). However, at typical docking ranges (1.0–2.0 m), SolvePnP often produces large errors exceeding 0.6 m (e.g., indices 1–17). In contrast, the proposed system maintains a consistent average error of approximately 1.18 m across all samples. These results confirm the superior accuracy and robustness of the proposed method for depth estimation in docking scenarios.

The mean error is computed using Equations (2) and (3).

(2) $${\mathrm{ME}}_{\mathrm{Proposed}}=\frac{1}{N}\sum_{i=1}^{N}\left|D_{\mathrm{LiDAR},i}-D_{\mathrm{Proposed},i}\right|$$

(3) $${\mathrm{ME}}_{\mathrm{SolvePnP}}=\frac{1}{N}\sum_{i=1}^{N}\left|D_{\mathrm{LiDAR},i}-D_{\mathrm{SolvePnP},i}\right|$$

Here, $D_{\mathrm{LiDAR},i}$ denotes the LiDAR-based ground-truth depth for the *i*-th sample, $D_{\mathrm{Proposed},i}$ is the depth predicted by the proposed method, $D_{\mathrm{SolvePnP},i}$ is the depth estimated by SolvePnP, and *N* is the total number of samples.

The raw data used to generate the graphs in Figure 12 are summarized in Table 4.

As shown in Table 4, SolvePnP yields relatively lower errors at short-range depths below approximately 1.0 m (e.g., indices 20–28, with errors ranging from 0.18 to 0.40 m). However, within the typical operational range for docking (1.0–2.0 m), SolvePnP produces large errors exceeding 0.6 m in most cases (e.g., indices 1–17). In contrast, the proposed system maintains a stable average error of approximately 1.18 m across all samples, producing predictions that closely match the LiDAR measurements. These results demonstrate that the proposed system outperforms SolvePnP in both accuracy and reliability, making it more suitable for docking applications.

### 4.7. Comparison of Orientation Prediction Performance Between the Proposed System and Existing Methods

**Figure 13:**
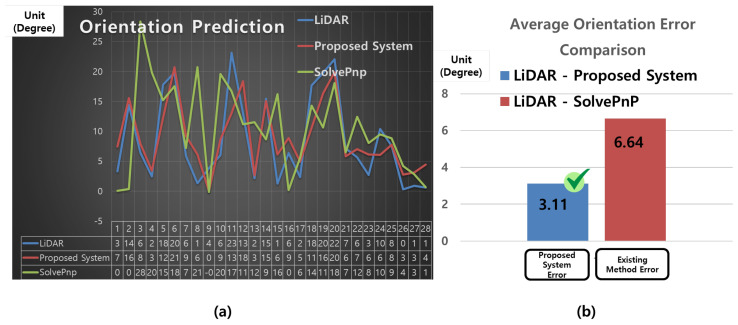
Comparison of orientation prediction performance between the proposed system and SolvePnP. (**a**) Time-series orientation prediction results (line graph): Orientation values (*y*-axis, in degrees) over sample indices (*x*-axis), comparing LiDAR ground truth (blue), proposed system (red), and SolvePnP (green). (**b**) Mean absolute error (MAE) comparison (bar graph): Average orientation prediction errors relative to LiDAR ground truth, with the proposed system (blue bar) and SolvePnP (red bar).

Figure 13a is a line graph based on the orientation prediction results presented in Table 5, showing the ground-truth values (A), the proposed system’s predictions (B), and the SolvePnP results (C). The x-axis represents the sample index, and the y-axis denotes the orientation in degrees (°). The blue line corresponds to the actual orientation measured by LiDAR (A), the red line indicates the orientation predicted by the proposed system (B), and the green line represents the orientation estimated by the SolvePnP method (C). As illustrated in the graph, the proposed system closely tracks the ground-truth orientation with stable accuracy, while the SolvePnP method exhibits large fluctuations and significant prediction errors.

Figure 13b presents a bar chart of the mean orientation errors (|A–B| and |A–C|) for both systems. The blue bar (“Proposed System Error”) indicates a mean error of 3.11°, whereas the red bar (“Existing Method Error”) shows a mean error of 6.64° for the SolvePnP method. These results quantitatively demonstrate that the proposed system achieves higher prediction accuracy and lower orientation error compared to the conventional approach.

The mean orientation error refers to the average absolute difference between the predicted and ground-truth orientation values for each sample. Here, $\theta_{\mathrm{LiDAR},i}$ denotes the ground-truth orientation measured by LiDAR at the *i*-th sample, $\theta_{\mathrm{Proposed},i}$ is the predicted orientation from the proposed system, and $\theta_{\mathrm{SolvePnP},i}$ is the orientation estimated by the SolvePnP method. *N* denotes the total number of samples. The mean orientation errors for the proposed system and SolvePnP are defined in Equations (4) and (5), respectively.

(4) $${\mathrm{ME}}_{\mathrm{Proposed}}=\frac{1}{N}\sum_{i=1}^{N}\left|\theta_{\mathrm{LiDAR},i}-\theta_{\mathrm{Proposed},i}\right|$$

(5) $${\mathrm{ME}}_{\mathrm{SolvePnP}}=\frac{1}{N}\sum_{i=1}^{N}\left|\theta_{\mathrm{LiDAR},i}-\theta_{\mathrm{SolvePnP},i}\right|$$

The graphs in Figure 13 are based on the data summarized in Table 5.

According to the results in Table 5, although SolvePnP yields small errors for some samples, it exhibits large deviations exceeding 10°, and in certain cases over 15°, in several instances (e.g., indices 3, 4, and 10). In contrast, the proposed system maintains most orientation errors within ±5°, resulting in a significantly lower overall mean error. These findings indicate that the proposed system offers more stable and reliable performance in orientation prediction.

### 4.8. Estimation of the Relative Lateral Distance Between the Robot and Charger

**Figure 14:**
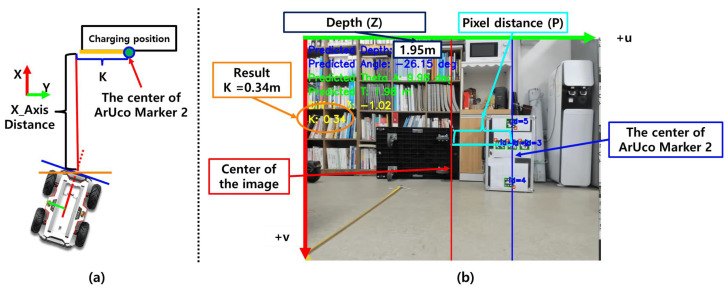
Triangulation-based estimation of lateral distance using monocular camera data.

In addition to depth and orientation estimation, accurately calculating the lateral distance is essential for precise docking alignment. In this study, ArUco Marker 2 is placed at the center of the docking position. As shown in Figure 14a, the lateral distance refers to the displacement along the y-axis of the robot’s coordinate frame, from the robot to the docking point, while the x-axis represents depth. In the diagram, the lateral distance is denoted by *K*, which quantifies how far the marker center deviates to the left or right from the robot’s forward direction.

The lateral distance *K* is computed based on the predicted depth *Z* and the lateral pixel displacement *P* between the center of the camera image and the center of ArUco Marker 2. In this study, the pinhole camera model is applied to project the pixel offset into a real-world lateral distance using the back-projection equation shown in Equation (6).

(6) $$K=\frac{Z\times P}{f}$$

Here, *f* denotes the horizontal focal length of the camera, *Z* is the depth value predicted by the regression model, and *P* represents the lateral pixel distance from the image center to the center of ArUco Marker 2. Figure 14b illustrates this relationship: the red line indicates the optical center of the camera, the blue line marks the center of Marker 2, and the cyan line denotes the horizontal pixel displacement *P* between them.

### 4.9. Docking Procedure

**Figure 15:**
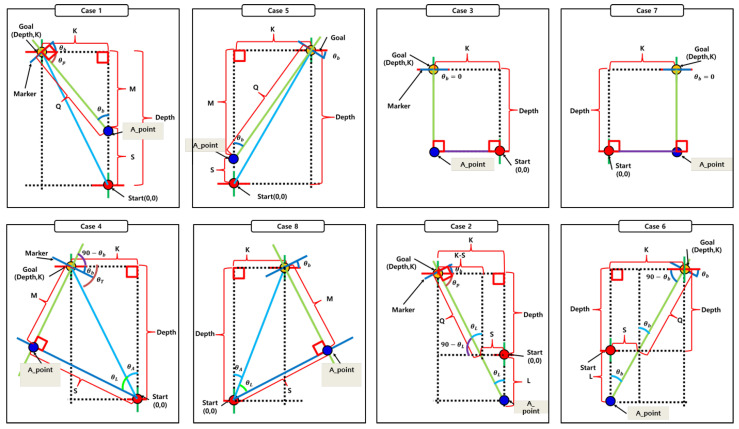
Eight predefined docking strategies categorized according to the robot’s relative pose to the docking station.

The proposed system performs docking by first estimating the relative position between the robot and the docking station using spatial information inferred from a regression model trained on ArUco markers. Based on the estimated position and orientation, one of eight predefined docking cases—illustrated in Figure 15—is selected to guide the docking maneuver.

**Figure 16:**
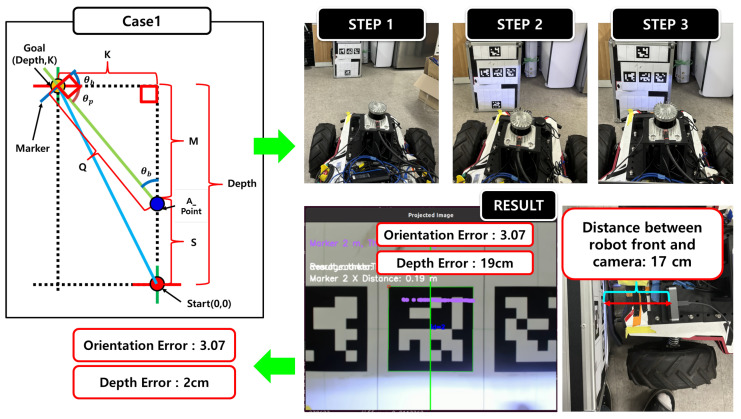
Two main procedures of the proposed system: regression model training and information acquisition for docking execution.

In the experiment, docking performance was evaluated using Case 1, as illustrated in Figure 16. In Step 1, the proposed model estimated the distance and orientation between the robot and the docking point, where the ideal condition corresponds to a distance error of 0 cm and an orientation error of 0°. In Step 2, the robot rotated by the predicted orientation to align perpendicularly with the docking target. In Step 3, proportional control (P-control) was applied to finely adjust the robot’s position and complete the docking maneuver.

After completing the docking, the final position error was measured using LiDAR as a reference. The results showed a distance error of 19 cm and an orientation error of 3.07°. Considering that the onboard camera is physically located 17 cm behind the front edge of the robot, the corrected final docking error is approximately 2 cm. These findings demonstrate that the proposed system can achieve high-precision docking even in real-world environments.

## 5. Conclusions

This study proposes a cost-effective autonomous docking system based on a monocular camera and ArUco markers, designing a regression-based method capable of accurately estimating depth, orientation, and lateral position without relying on expensive LiDAR sensors.

To validate system performance, comparative experiments were conducted against SolvePnP and LiDAR-based measurement methods. The proposed system recorded an average distance prediction error of 1.18 cm, significantly lower than that of SolvePnP (58.54 cm), and demonstrated accuracy comparable to LiDAR-based measurements. For orientation prediction, it achieved an average error of 3.11°, outperforming the conventional method (6.64°) in angular precision.

These results indicate that the regression model effectively learns the nonlinear spatial relationships from variations in the size and position of ArUco markers. Notably, SolvePnP suffers from rapidly increasing errors when markers are viewed obliquely or from a distance due to its reliance on projective geometry, whereas the proposed method maintains robust performance across various distance and orientation conditions.

Furthermore, real-world tests of various docking scenarios showed an average final docking position error of approximately 2 cm, sufficiently meeting the precision requirements of logistics and industrial environments.

Despite using only a monocular camera, the system achieved performance similar to LiDAR-based systems, demonstrating its potential as a practical alternative considering cost and installation space constraints.

However, the current system requires precise camera-sensor calibration and stable lighting conditions, factors that may affect performance depending on the application environment. Future work will focus on improving generalization under varying illumination and environmental conditions, as well as enhancing online learning methods and real-time processing to optimize docking speed and robustness.

## Figures and Tables

**Table 1:** Performance comparison of regression models for depth prediction. The random forest model, highlighted in yellow, indicate the best performance in depth prediction.

Model Name	Best Test MSE	Best Test $R^{2}$
Linear Regression	0.0233	0.8350
Ridge Regression	0.0255	0.8192
Lasso Regression	0.0352	0.7505
Decision Tree	0.0014	0.9901
Random Forest	0.0009	0.9937
SVR	0.0120	0.9148
Gradient Boosting	0.0017	0.9880

**Table 2:** Best-performing regression model for each depth segment based on lowest MSE (*N*: number of samples).

Depth Range (25–140 cm)					Depth Range (145–250 cm)				
Distance	N	Regression Model	MSE	$R^{2}$	Distance	N	Regression Model	MSE	$R^{2}$
25–30 cm	69	Gradient Boosting	0.0099	0.9847	135–140 cm	705	Random Forest	3.5072	0.8887
30–35 cm	276	Gradient Boosting	0.0814	0.9773	140–145 cm	598	Random Forest	1.7674	0.9393
35–40 cm	335	Random Forest	0.0932	0.9343	145–150 cm	396	Gradient Boosting	1.3094	0.9430
40–45 cm	431	Decision Tree	0.0814	0.9770	150–155 cm	458	Random Forest	1.9674	0.9341
45–50 cm	429	Gradient Boosting	0.3576	0.9854	155–160 cm	193	Gradient Boosting	3.0796	0.9426
50–55 cm	428	Gradient Boosting	0.4795	0.9785	160–165 cm	138	Decision Tree	0.3289	0.9897
55–60 cm	366	Random Forest	0.2585	0.9799	165–170 cm	117	Decision Tree	1.0112	0.9505
60–65 cm	460	Random Forest	1.6030	0.8857	170–175 cm	101	Decision Tree	2.4376	0.5530
65–70 cm	577	Random Forest	0.5087	0.9754	175–180 cm	23	Gradient Boosting	12.7052	0.5293
70–75 cm	494	Decision Tree	0.4155	0.9578	180–185 cm	21	Decision Tree	0.0257	0.4752
75–80 cm	442	Random Forest	0.5615	0.9880	185–190 cm	18	Random Forest	0.2498	0.9982
80–85 cm	338	Gradient Boosting	0.6338	0.9779	190–195 cm	49	Ridge Regression	1.4240	0.8733
85–90 cm	369	Random Forest	0.3977	0.9930	195–200 cm	83	Random Forest	2.9834	0.7437
90–95 cm	469	Random Forest	0.9296	0.9768	200–205 cm	63	Ridge Regression	2.1568	0.8631
95–100 cm	433	Random Forest	0.7588	0.9839	205–210 cm	61	Gradient Boosting	0.5281	0.9918
100–105 cm	957	Random Forest	1.4792	0.9793	210–215 cm	93	Gradient Boosting	2.1457	0.9265
105–110 cm	982	Random Forest	0.7898	0.9859	215–220 cm	49	Random Forest	0.2762	0.9541
110–115 cm	853	Random Forest	1.3915	0.9734	220–225 cm	9	Linear Regression	0.0181	0.9988
115–120 cm	977	Random Forest	1.2175	0.9818	225–230 cm	7	Linear Regression	0.2861	0.0264
120–125 cm	934	Random Forest	2.0215	0.9733	230–235 cm	8	Random Forest	0.9308	0.1611
125–130 cm	703	Random Forest	2.6560	0.9523	235–240 cm	10	Linear Regression	1.3492	0.3258
130–135 cm	909	Random Forest	1.9862	0.9543	240–245 cm	47	Decision Tree	1.1248	0.9142
					Avg	–	–	1.5185	0.915

**Table 3:** Comparison of mean squared error (MSE) among seven regression models across five selected distance intervals. The lowest MSE values, highlighted in yellow, indicate the best-performing model for each interval.

Model	140–145 cm	145–150 cm	150–155 cm	155–160 cm	160–165 cm
Decision Tree	1.8441	1.5568	1.9931	5.8053	0.3289
Gradient Boosting	1.7674	1.3094	2.0993	3.0796	0.6183
Lasso Regression	27.3481	26.4010	26.9940	27.5812	19.4641
Linear Regression	27.5862	26.7054	27.2161	264.1204	19.8546
Random Forest	1.8173	1.4074	1.9674	3.2851	0.4118
Ridge Regression	20.9879	20.4370	21.0114	17.7053	15.0084
SVR	28.9687	28.7546	33.2085	55.4888	30.5262

**Table 4:** Comparison of distance measurement performance between the proposed system and SolvePnP. Left: Index 1–14, Right: Index 15–28. A: LiDAR (m), B: Proposed (m), C: SolvePnP (m), D: |A–B| (m), E: |A–C| (m).

Index 1–14						Index 15–28					
Idx	A	B	C	D	E	Idx	A	B	C	D	E
1	1.04	1.07	1.59	0.03	0.55	15	1.50	1.48	2.26	0.02	0.76
2	1.62	1.62	2.52	0.00	0.90	16	1.40	1.38	2.15	0.02	0.75
3	1.54	1.52	2.28	0.02	0.74	17	1.21	1.21	1.82	0.00	0.61
4	1.39	1.39	2.11	0.00	0.72	18	1.15	1.15	1.72	0.00	0.57
5	1.33	1.33	1.96	0.00	0.63	19	1.08	1.09	1.57	0.01	0.49
6	1.18	1.17	1.78	0.01	0.60	20	0.96	0.96	1.36	0.00	0.40
7	1.06	1.07	1.62	0.01	0.56	21	0.72	0.72	1.10	0.00	0.38
8	1.19	1.20	1.79	0.01	0.60	22	0.63	0.63	0.97	0.00	0.34
9	1.42	1.42	2.18	0.00	0.76	23	0.61	0.60	0.90	0.01	0.29
10	1.44	1.44	2.23	0.00	0.79	24	0.44	0.44	0.62	0.00	0.18
11	2.24	2.13	3.34	0.11	1.10	25	0.40	0.41	0.58	0.01	0.18
12	2.17	2.13	3.23	0.04	1.06	26	0.37	0.37	0.55	0.00	0.18
13	1.83	1.81	2.81	0.02	0.98	27	0.39	0.39	0.60	0.00	0.21
14	1.65	1.66	2.47	0.01	0.82	28	0.44	0.44	0.68	0.00	0.24
						Avg	–	–	–	0.01	0.59

**Table 5:** Comparison of orientation prediction performance between the proposed system and SolvePnP based on LiDAR measurements. Left: Index 1–14, Right: Index 15–28. A: LiDAR (°), B: Proposed (°), C: SolvePnP (°), D: |A–B| (°), E: |A–C| (°).

Index 1–14						Index 15–28					
Idx	A	B	C	D	E	Idx	A	B	C	D	E
1	3.32°	7.45°	0.08°	4.13°	3.24°	15	1.29°	6.12°	16.22°	4.83°	14.93°
2	14.44°	15.59°	0.36°	1.15°	14.08°	16	6.40°	8.91°	0.172°	2.51°	6.23°
3	6.44°	7.91°	28.37°	1.47°	21.93°	17	2.38°	4.91°	5.62°	2.53°	3.24°
4	2.47°	3.46°	19.84°	0.99°	17.37°	18	17.64°	10.57°	14.29°	7.07°	3.35°
5	17.81°	12.19°	15.21°	5.62°	2.60°	19	19.78°	16.32°	10.65°	3.46°	9.13°
6	19.83°	20.74°	17.53°	0.91°	2.30°	20	22.03°	19.87°	18.09°	2.16°	3.94°
7	5.92°	9.26°	7.20°	3.34°	1.28°	21	7.17°	5.85°	6.52°	1.32°	0.65°
8	1.32°	6.14°	20.75°	4.82°	19.43°	22	5.63°	7.02°	12.42°	1.39°	6.79°
9	3.88°	0.00°	−0.12°	3.88°	4.00°	23	2.74°	6.13°	8.03°	3.39°	5.29°
10	6.01°	8.68°	19.59°	2.67°	13.58°	24	10.40°	6.05°	9.50°	4.35°	0.90°
11	23.11°	13.03°	16.70°	10.08°	6.41°	25	7.55°	7.73°	8.83°	0.18°	1.28°
12	13.05°	18.41°	11.18°	5.36°	1.87°	26	0.29°	2.79°	4.23°	2.50°	3.94°
13	2.20°	2.67°	11.57°	0.47°	9.37°	27	0.90°	3.13°	2.84°	2.23°	1.94°
14	15.43°	15.09°	8.65°	0.34°	6.78°	28	0.64°	4.43°	0.68°	3.79°	0.04°
						Avg	–	–	–	3.11°	6.64°

## Data Availability

The raw data supporting the conclusions of this article will be made available by the authors on request.
